# Creation and Implementation of a Mastery Learning Curriculum for Emergency Department Thoracotomy

**DOI:** 10.5811/westjem.2020.5.46207

**Published:** 2020-08-24

**Authors:** Danielle T. Miller, Hashim Q. Zaidi, Priyanka Sista, Sarah S. Dhake, Matthew J. Pirotte, Abra L. Fant, David H. Salzman

**Affiliations:** * Stanford University School of Medicine, Department of Emergency Medicine, Palo Alto, California; † University of Chicago School of Medicine, Department of Emergency Medicine, Chicago, Illinois; ‡ Northwestern University Feinberg School of Medicine, Department of Emergency Medicine, Chicago, Illinois; § NorthShore University Health System, Department of Emergency Medicine, Chicago, Illinois; ¶ Vanderbilt University School of Medicine, Department of Emergency Medicine, Nashville, Tennessee

## Abstract

**Introduction:**

Emergency department thoracotomy (EDT) is a lifesaving procedure within the scope of practice of emergency physicians. Because EDT is infrequently performed, emergency medicine (EM) residents lack opportunities to develop procedural competency. There is no current mastery learning curriculum for residents to learn EDT. The purpose of this study was to develop and implement a simulation-based mastery learning curriculum to teach and assess EM residents’ performance of the EDT.

**Methods:**

We developed an EDT curriculum using a mastery learning framework. The minimum passing standard (MPS) for a previously developed 22-item checklist was determined using the Mastery Angoff approach. EM residents at a four-year academic EM residency program underwent baseline testing in performing an EDT on a simulation trainer. Performance was scored by two raters using the checklist. Learners then participated in a novel mastery learning EDT curriculum that included an educational video, hands-on instruction, and deliberate practice. After a three-month period, residents then completed initial post testing. Residents who did not meet the minimum passing standard after post testing participated in additional deliberate practice until mastery was obtained. Baseline and post-test scores, and time to completion of the procedure were compared with paired t-tests.

**Results:**

Of 56 eligible EM residents, 54 completed baseline testing. Fifty-two residents completed post-testing until mastery was reached. The minimum passing standard was 91.1%, (21/22 items correct on the checklist). No participants met the MPS at the baseline assessment. After completion of the curriculum, all residents subsequently reached the MPS, with deliberate practice sessions not exceeding 40 minutes. Scores from baseline testing to post-testing significantly improved across all postgraduate years from a mean score of 10.2/22 to 21.4/22 (p <0.001). Mean time to complete the procedure improved from baseline testing (6 minutes [min] and 21 seconds [sec], interquartile range [IQR] = 4 min 54 sec - 7 min 51 sec) to post-testing (5 min 19 seconds, interquartile range 4 min 17sec - 6 min 15 sec; p = 0.001).

**Conclusion:**

This simulation-based mastery learning curriculum resulted in all residents performing an EDT at a level that met or exceeded the MPS with an overall decrease in time needed to perform the procedure.

## INTRODUCTION

The emergency department thoracotomy (EDT) is a rare, lifesaving procedure that is within the scope of practice of emergency physicians.^1^,^2^ EDT is a complex procedure that involves opening the thoracic cavity to intervene on critical injuries to the heart and other thoracic structures. Due to the infrequency of clinical exposure, studies of emergency medicine (EM) residents suggest minimal opportunities to develop procedural competency in EDT.^3^–^7^ Despite the infrequent presentation, EM residents must have adequate training to achieve the skills required to competently perform this emergent procedure. Unfortunately, there is a paucity of literature describing the ideal teaching approach.

Early teaching modalities have included written and computer modules to teach and assess trainees.^8^,^9^ However, studies of these teaching modalities have concluded that tactile performance is a necessary component of developing procedural competency.^8^ Cadaveric or porcine models have been explored for establishing proficiency; however, the expense of the models and the need for repetitive deliberate practice to ensure competency have made these modalities cost-prohibitive for education on a widespread basis.^3^,^5^,^7^ High- fidelity simulation models are increasingly being employed to allow for repetitive practice; however, no competency-based curriculum currently exists for EDT.^10^–^13^

Mastery learning is a well-regarded, reliable, and highly effective competency-based education approach within health professions education. Its core tenants dictate that trainees must achieve an a priori-defined level of high proficiency in a given instructional unit with little to no variation prior to proceeding to the next unit.^14^,^15^ Simulation-based mastery learning involves repetitive simulated performance of the intended cognitive or psychomotor skills coupled with rigorous personalized and informative feedback, with the goal of achieving mastery of the subject. This process entails establishing a minimum passing standard (MPS), baseline testing of the target skills on simulated models, deliberate practice of target skills, and continued practice with further testing until the MPS is reached.^16^–^18^ Simulation-based mastery learning has been used in graduate medical education training to provide procedural exposure in a safe environment, allow for deliberate practice, and evaluate procedural competency.^19^–^22^ Simulation-based mastery learning has been found to be superior to non-mastery instruction in procedural success rates, procedure time, and complication rates.^17^,^18^

This study had several objectives. The first was to develop and implement a simulation-based mastery learning curriculum for EDT. Second was to establish a MPS for a previously developed 22-item checklist for use in this mastery curriculum.^23^ Third was to determine whether this mastery learning curriculum could result in achievement of the MPS by all participants. Fourth was to compare baseline and final post-test performance on checklist items and time to perform an EDT in a simulated environment. Fifth was to determine participant satisfaction with the curriculum.

**Population Health Research Capsule**

What do we already know about this issue?

Emergency department thoracotomy (EDT) is a lifesaving procedure, but emergency medicine (EM) residents lack opportunities to develop procedural competency.

What was the research question?

Can a simulation-based mastery learning curriculum on EDT improve resident procedural skills?

What was the major finding of the study?

The simulation-based mastery learning curriculum resulted in all residents performing an EDT at mastery level.

How does this improve population health?

The simulation-based mastery learning curriculum can be used for EM residents to gain competency in this rare but life-saving procedure.

## METHODS

### Study Setting and Population

This was a prospective cohort study of EM residents participating in a mastery learning curriculum for EDT. The study was conducted at a four-year academic EM residency training program from July 2018–June 2019. All participants were EM residents. Residents included postgraduate year one (PGY1) to PGY4 levels. Four residents involved in the study design, checklist creation, and session facilitation were excluded from participation. Participants were informed about the study and invited to participate voluntarily. This study was reviewed by the institutional review board at Northwestern University and deemed to be exempt.

### Standard Setting

A MPS for the previously published checklist^23^ was established by an expert panel using the mastery Angoff method^24^. A panel of 17 physicians – 15 emergency physicians and two trauma surgeons with experience performing and teaching the procedure – were recruited to serve as judges for the standard-setting process. Judges were asked to estimate the percentage of well-prepared learners who would perform each checklist item correct at the completion of training. A “well-prepared” learner was defined as a resident who could safely and successfully perform the procedure without supervision in clinical practice. Judges completed an electronic form between May–July 2018 using Qualtrics (Qualtrics LLC, Provo, UT).

### Baseline Assessment

**Figure 1:**
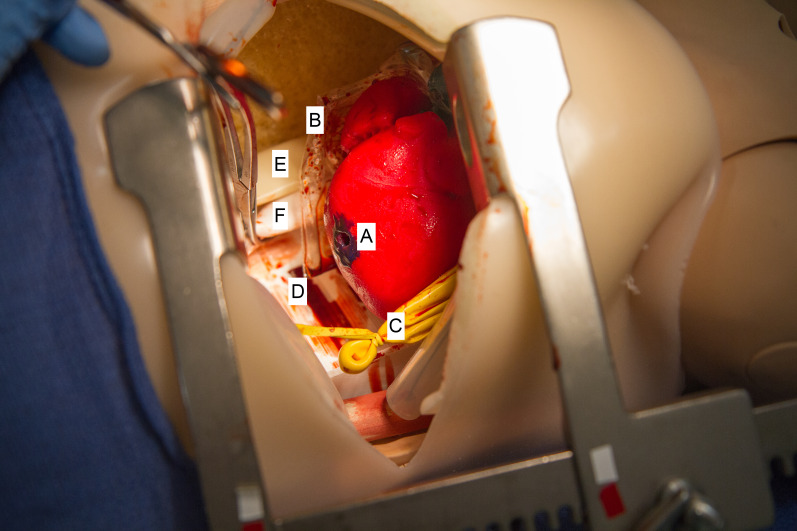
Image of the thoracotomy simulation trainer, with views of the interior chest structures: A) heart with cardiac wound; B) incised pericardium; C) inferior left lung; D) posterior ribs; E) esophagus; and (F) aorta.

All 56 eligible PGY1-4 EM residents were invited to participate in the curriculum. EM residents were asked to quantify the number of EDTs performed in the clinical environment and the simulated environment prior to baseline assessment. During a three-month period, participants were scheduled to complete a baseline assessment. A novel simulated thoracotomy model created by Northwestern Simulation was used in assessment and teaching (Figure 1). This model featured realistic, three-dimensional printed anatomical features including skin and subcutaneous tissue, ribs, lungs, heart, pericardium, phrenic nerve, blood, aorta, esophagus, and spine. In baseline testing, each resident was presented with a clinical scenario of a patient with a penetrating trauma who had just lost vital signs immediately prior to arrival to the ED and was asked to perform an EDT on the simulated model. Performance was recorded using the checklist and was completed by two raters. A total of seven raters (four women, three men) were trained for these sessions. All raters were EM trained and included three EM faculty and four EM senior residents (PGY3 or PGY4). Residents who participated as raters were not included in mastery learning data outcomes. Sessions were audio and video recorded and made available to the raters if needed for review. Sessions were timed. Neither the raters nor the participants knew the MPS.

### Curriculum Intervention

In the subsequent three months after completion of the baseline simulation assessments, residents participated in educational sessions. Components of the curriculum included a detailed instructional procedure video, individualized instruction through skills stations, and deliberate practice performing a simulated EDT with feedback. Learners did not see or have access to the checklist throughout the curriculum.

An EDT procedural video was created specifically for this curriculum by the Northwestern Simulation Lab in conjunction with the Northwestern Innovations Lab (Appendix 2). The educational video was created by a team of emergency and trauma physicians and contained 11 sections: Overview; Indications; Contraindications; Anatomy; Equipment; Preparation; Procedure; Troubleshooting; Aftercare; Complications; and References. Learners were assigned to watch the video individually prior to the practice sessions. After watching the video, residents participated in a 20-minute, individual hands-on practice session. Of the seven checklist raters, six (three women, three men) were trained as facilitators for these sessions. The 20-minute sessions were divided into three stations, relating to specific actions to perform an EDT.

In the first station, the facilitator reviewed the instruments used in performing an EDT. This included both a review of all instruments and practice with assembling the rib spreader. The second station included identification of anatomic structures on the simulated model, along with instruction on the control of cardiac hemorrhage via Foley catheter insertion, staple, or suturing with pledgets. The third station included identification of the aorta and esophagus on the simulated model, an explanation on how to identify each structure respectively, and a demonstration of how to cross-clamp the aorta. Trainees were then given the opportunity to practice the entire EDT procedure with real-time feedback on their performance and were allowed to come to additional sessions for deliberate practice as desired. To accommodate resident scheduling, the deliberate practice sessions occurred during a three-month period from completion of baseline testing.

### Post-testing

During a three-month period following completion of the deliberate practice sessions, residents underwent initial post-testing. Each resident was asked to perform an EDT on the simulated model. Performance was assessed using the same checklist and was completed by two raters. Sessions were video recorded and made available to the raters if needed for review. The same seven raters for baseline testing completed post-testing sessions. Residents who did not reach MPS at initial post-testing returned for additional deliberate practice at a later date. During the subsequent session, residents were informed of missed or incorrect steps during their initial assessment to direct their additional deliberate practice. Any participant not meeting or exceeding MPS continued with deliberate practice and testing until the MPS was met. After achievement of mastery, residents completed a post-curriculum survey. The post-curriculum survey used a Likert scale 1–5 for the estimation of self-efficacy in performing EDT after the curriculum intervention and desirability of future inclusion of the curriculum in residency training.

### Statistical Analysis

We analyzed score differences on the baseline performance and post-curriculum intervention using paired t-tests, Stata version 14 (StataCorp LLC, College Station, TX). Within-group differences for PGY1-4 from baseline performance to post-testing were also analyzed using paired t-tests. We analyzed time to completion of the procedure from baseline testing to post-testing using paired t-tests. The pre-curriculum and post-curriculum surveys were analyzed using central tendency metrics.

## RESULTS

**Figure 2:**
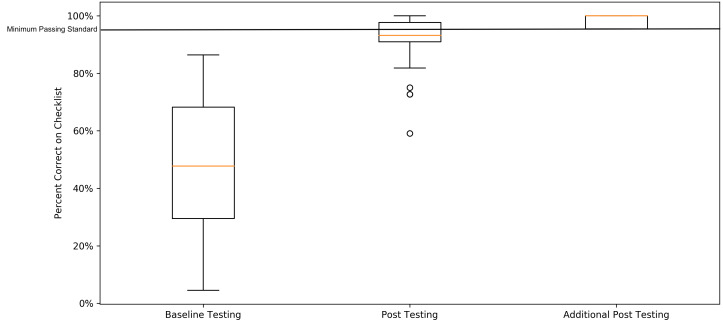
Baseline testing, post-testing, and additional post-testing scores of emergency medicine residents on the emergency department thoracotomy procedural checklist with a line demarcating the minimum passing standard.

The minimum passing standard was calculated to be 91.1%. To meet or exceed this threshold, the learner needed to perform 21 of 22 checklist items correctly. Of 56 eligible residents, 54 completed baseline testing (Table 1). Two residents were unable to complete baseline testing due to scheduling conflicts. Fifty-two residents completed post- testing until mastery was reached (Table 2). In pre-curriculum survey data, 9.6% of participants had performed an EDT in the clinical environment and 22.6% in the simulated environment. No participants met the MPS at the baseline assessment. After completion of the curriculum, all residents subsequently reached the MPS (Figure 2).

Of the 52 residents who completed post-testing, 31 passed on initial post-curriculum testing with the remaining 21 achieving the MPS after additional deliberate practice (Table 2). The amount of deliberate practice time did not exceed 40 minutes. Comparison of mean scores from baseline testing to final post-testing across all PGY years significantly improved from average raw score of 10.2/22 (standard deviation [SD] = 4.8), to 21.4/22 (SD = 0.6, t(52) = 16.7, p <0.001). Comparison of the mean percentage of items correct on the checklist from baseline to initial post-testing was also significant (average raw score of 10.1/22, SD = 4.8 to 20.2/22, SD = 1.7, t[52] = 15.5, p <0.001). Average time to complete the procedure in baseline testing (M = 6 minutes [min] and 21 seconds [sec], interquartile range [IQR] = 4 min 54 sec - 7 min 51 sec) compared to final post-testing (M = 5 min 19 sec, IQR = 4 min 17 sec - 6 min 15 sec) was significant (t [52] =3.4, p = 0.001).

Participants reported an improvement in confidence for performing the procedure (median grade of 4 on 5-point Likert scale). Participants reported the desire for this curriculum to be included in the future curriculum for the residency (median grade of 5 on 5-point Likert scale).

## DISCUSSION

Our study demonstrates a simulated-based mastery learning curriculum can effectively develop EDT skills in EM residents. To our knowledge, this is the first mastery learning curriculum to teach EDT. This curriculum adds another procedure to the list where mastery learning can function as an educational strategy to improve baseline procedural skills in residents, as seen with other mastery learning curricula, such as central venous cannulation, lumbar puncture, and thoracentesis.^19^,^21^,^22^ The data obtained during the baseline assessment, where no resident was able to achieve the MPS, provides supporting evidence that residents have limited experience and instruction on this procedure and that current educational approaches are insufficient to ensure graduating residents are able to perform this critically important emergent procedure.

All residents who participated in the curriculum were able to achieve the MPS within one or two 20-minute sessions of deliberate practice. The mean score on the procedural checklist after the curriculum intervention improved for all PGY levels (p<0.001). Similar to other mastery learning curricula, the outcomes demonstrate a uniform high performance with minimal variability of performance. The average time to perform the procedure also improved by an average of 62 sec (from 6 min 21 sec to 5 min 19 sec, p = 0.001). Considering that EDT is performed on patients in or very near cardiac arrest, the improvement in time to perform this procedure was an important outcome. The significant improvement of scores from baseline to initial post-testing also demonstrates that improvement in skills can be achieved, although progression to ensure all participants meet mastery standards requires additional deliberate practice. Our analysis also shows that self-reported resident confidence in performing an EDT after the curriculum intervention was high (median 4 on 5-point Likert scale). These findings are similar to previous findings of increased confidence in residents after mastery learning training in other procedures.^19^,^21^

Steps in baseline testing that were rarely performed correctly included ensuring all instruments were present (mean percent of residents performing correctly 9.3%); maintaining sterility (22.2%); gathering equipment (29.6%); controlling cardiac hemorrhage (29.6%); and cross-clamping the aorta (29.6%). These results are similar to our previous study in which a pilot group of general surgery and EM residents and attendings performed an EDT on the simulation trainer and were evaluated with the checklist; those who had not performed an EDT in the clinical environment had lower mean scores on ensuring all instruments were present, maintaining sterility, and gathering equipment.^23^ In the pilot study, those who had not performed an EDT in the clinical environment also on average performed worse on all steps involved in controlling cardiac hemorrhage from incising the pericardium, to delivering the heart, to controlling hemorrhage via Foley catheter, suture, or pledgets.

Mastery learning is an ideal educational strategy for teaching EDT, as clinical experience alone is clearly not sufficient for training. In addition, our data shows that previous experience alone does not predict procedural competency as none of these residents achieved MPS in baseline testing. While previous studies have created curricula to teach this procedure, none have been mastery based. Bohnen et al created an EDT curriculum for surgical residents.^12^ This pilot study included eight expert and six novice surgeons performing an EDT on a simulation model. While this study created a checklist, it focused on five broad tasks for performing the procedure: 1) opening chest/rib spreader utilization; (2) pericardiotomy/cardiac repair; (3) open cardiac massage; (4) clamping aorta; and (5) control of pulmonary hilum. Residents were evaluated using a surgical assessment tool, the Objective Structured Assessment of Technical Skills, which has not been validated in EM and focuses on (1) surgical technique, (2) general skills, and (3) global rating. This checklist and curriculum are not easily translated to EM.

Our approach provides a more detailed checklist for the procedure, and while initially designed with a focus for EM residents, the checklist and curriculum could be used by any learner who needs to learn how to perform an EDT. Additionally, while two previous studies have created curriculum to teach EDT, neither has assessed for competence or mastery learning.^12^,^13^ Both have been small pilot studies showing improvement in confidence performing the procedure after a curriculum intervention but not mastery of the procedure.

The video created by this curriculum is also an additional resource for procedural teaching. Previous research has shown that videos for procedural teaching can be an effective modality for learning. For example, a previous study by Saun et al demonstrated that a *New England Journal of Medicine* video on the procedure of chest tube insertion was as effective as a video-recorded didactic for teaching the knowledge and technical skills for chest tube insertion, with participants expressing high satisfaction with the new modality.^25^ Current videos on the procedure of EDT often have poor visualization of anatomic structures, or often have limited instruction on when and how to perform the procedure.^26^–^28^ The EDT video that we created for this curriculum allows for proper visualization of the anatomic components of the procedure. Additionally, this video contains key instruction on indications, contraindications, anatomy, equipment, troubleshooting, complications, and aftercare, which, to our knowledge, current videos do not fully encompass.

Arguments against mastery learning have often noted that mastery learning compared to non-mastery learning requires more time.^17^ The estimated time requirement for this program included 18 four-hour sessions. This time was divided into five days of baseline testing, eight days of deliberate practice, and five days of post- testing. For those who did not meet MPS on initial post-testing, an average of 20 of additional deliberate practice and 10 minutes of retesting were required, with no learner exceeding 40 minutes. While our curriculum included individualized instruction for 20 minutes with a facilitator leading a learner through three stations, the curriculum could be altered to decrease time required of facilitators by grouping residents during these stations. Additionally, during analysis of the baseline assessment, we found several steps with particularly low correct performance (ensuring all instruments were present, maintaining sterility, gathering equipment, controlling cardiac hemorrhage, and cross-clamping the aorta). If residency programs have limited time and resources to perform this mastery learning curriculum, these experiences could guide resource allocation for practice sessions.

## LIMITATIONS

This study was conducted at a large urban, academic four-year EM residency program in the United States, and thus may not be generalizable. This study also was conducted at an institution with access to a simulated model to provide this educational intervention to residents. We did not assess for resident performance of EDT in a patient care environment, and thus we cannot comment on translation of skills into the clinical environment. Future work could potentially use the checklist in a video-recorded clinical environment to assess for competency. Additionally, given this is a rare procedure with high mortality rates, we were unable to assess patient-centered outcomes for this educational intervention, including patient morbidity and mortality. Furthermore, we were unable to determine retention of this skill due to limitations of funding and academic calendar scheduling. Ideally, we would have completed retention assessment six months to one year following achievement of mastery to inform whether additional practice is needed to maintain skills necessary to perform an EDT to a mastery level. Finally, more studies are needed to explore whether additional teaching modalities are as effective in teaching this procedure.

## CONCLUSION

In conclusion, this study demonstrates that a simulation-based, mastery learning curriculum improves performance of residents in simulated EDT. This curriculum can be used for residents to gain competency in this rare but life-saving procedure.

## Supplementary Information







## Figures and Tables

**Table 1:** Demographic data of emergency medicine resident participants in thoracotomy simulation training.

Characteristic	Residents (n = 54)
Male	36 (66.7%)
Female	18 (33.3%)
PGY1	15 (27.8%)
PGY2	15 (27.8%)
PGY3	14 (25.9%)
PGY4	10 (18.5%)

*PGY*, postgraduate year.

**Table 2:** Baseline testing, post-testing, and additional post-testing scores on a 22-item checklist of emergency medicine residents on the procedure of emergency department thoracotomy.

	Baseline testing			Post-testing			Additional post-testing		

	n=54	Mean checklist score out of 22 (SD)	Number met MPS (n)	n=52	Mean checklist score out of 22 (SD)	Number met MPS (n)	n=21	Mean checklist score out of 22 (SD)	Number met MPS (n)
PGY1	15	7.3(3.9)	0	15	19.5(1.8)	5	10	21.5(0.5)	10
PGY2	15	8.2 (5.1)	0	15	20.5(2.2)	11	4	21.6(0.6)	4
PGY3	14	13.7(3.1)	0	14	20.2(1.5)	8	6	21.8 (0.4)	6
PGY4	10	13.4 (2.5)	0	8	20.9(0.6)	7	1	21 (-)	1

*SD*, standard deviation; *MPS*, minimum passing standard; *PGY*, postgraduate year.
