# Supplementary material for: Creation and Implementation of a Mastery Learning Curriculum for Emergency Department Thoracotomy
Source: West J Emerg Med. 2020 Aug 24;21(5):1258–65. doi: 10.5811/westjem.2020.5.46207 (PMC7514402; doi:10.5811/westjem.2020.5.46207)
Supplement: Supplementary file 1 [file wjem-21-1258-s001.pdf]

Participant Name: ________________________________ SCORE / TIME: _________

Department: EM / Surgery Evaluator: ____________________

Level of Training: Attending / Fellow / Resident (PGY: ______)

<u>For Evaluators</u>:
Please instruct the candidates with the following prompt: "You just received an EMS call. The patient sustained a penetrating trauma to the chest and EMS reports that the patient just lost vital signs. ETA 3minutes. At this time, the decision is made to prep for an emergent thoracotomy. Please gather all necessary materials and perform an emergent thoracotomy from start to finish and verbalize the actions you are performing. You will be told once your patient has arrived."

Please allow the participant to perform steps 1-3. Once steps 1-3 are complete to the participant's satisfaction, please inform the participant "Your patient has arrived" and start the timer. Stop the timer once the participant has initiated cardiac massage or the participant wishes to end the scenario. Please mark below whether the candidate performs the action as "CORRECT" or "INCORRECT" in the space provided. Please note, some actions have a correct or incorrect order to be performed and explanations for correct performance of each item are included where applicable.

	**CORRECT**	**INCORRECT**
**1. Don full PPE**<br>Correct: Must wear gown, gloves, mask, and eye protection.		
**2. Gather equipment**<br>Correct: Must include thoracotomy tray, scalpel, sterile towels or drape, betadine or chlorhexidine.		
**3. Ensure all required instruments are in the thoracotomy tray**<br>Correct: Must verbalize the supplies of scalpel, scissors (Metzenbaums/Mayo), rib spreaders (Tuffier), forceps (Pickups), vascular clamps (Debakey/Satinsky), needleholder, sternal or bone saw/cutters (Bone mallet/Lebsche knife/Gigli saw).<br><br>*Evaluator prompt if candidate does not verbalize items: "What items do you require to perform this procedure?"*		
**4. Assemble the rib spreader prior to incision**<br>Correct: Places the ratchet apparatus between the two arms of the spreader and ensures that the spreader will open and close. When finished assembling, leaves in the fully closed position. Performs this step prior to incision.		
**5. Position the patient**<br>Correct: Raises and abducts the arm above the patient's head to expose the left chest.		
**6. Prepare chest prior to incision**<br>Correct: Uses betadine or chlorhexidine and performs the action prior to incision.		

	CORRECT	INCORRECT
**7. Incision**<br>Correct: Makes an incision at the level of the 4th or 5th intercostal space starting at the sternum extending posteriorly to nearly the level of the bed just under nipple for male, under the breast fold for female.<br><br>*Evaluator prompt: "Describe your approach. Please be specific." (Must include specific intercostal space, and length of incision e.g. sternum all the way lateral to mid/posterior axillary line nearly to the level of the bed).*		
**8. Extend incision to expose and separate ribs/intercostals**<br>Correct: Uses either the scissors or scalpel to separate the intercostal muscles, cutting along the length of the incision.<br><br>*Evaluator may prompt if the candidate has cut entirely through the skin: "How would you have approached a similar patient if you hadn't been able to cut through all of the layers and gain access to the thoracic cavity?"*		
**9. Manually spread ribs**<br>Correct: Uses hands to initiate rib spreading for placement of the rib spreader.<br><br>*Evaluator Action: Begin manually ventilating at head of bed. If the candidate requests right mainstem ventilation cease ventilating and verbalize "The patient continues to be ventilated with right main-stem intubation."*		
**10. Insert rib spreader**<br>Correct: Proper insertion of the rib spreader into the thoracic cavity with the rachet apparatus downwards towards the bed.		
**11. Open rib spreader**<br>Correct: Rib spreader is opened to allow access to the thoracic cavity and visualization of the heart.		
**12. Identify the heart and pericardial sac, moving the lung if needed**<br><br>*Evaluator may prompt the candidate to show them the heart and relevant structures.*		
**13. Identify the phrenic nerve prior to pericardiotomy**<br><br>*Evaluator may prompt candidate to identify relevant structures prior to pericardiotomy.*		
**14. Lift pericardium with pickups**		

	CORRECT	INCORRECT
**15. Incise the anterior pericardium**<br>Correct: Incision is from cephalad to caudal and parallel to but not involving the phrenic nerve. May be incised with scalpel or scissors.<br><br>*Evaluator Prompt: "Please detail how you would incise the pericardium" Must include direction (eg head to toe, cephalad to caudal, etc) and mention identifying or avoiding the phrenic nerve.*<br>*Evaluator may prompt requesting direction or relevant structures.*		
**16. Deliver the heart through the pericardiotomy and evacuate any hemopericardium**		
**17. Identify any cardiac injury**<br>Correct: Verbalizes that they do or do not see any visible cardiac injury (i.e. there is a laceration or puncture/hole/GSW).<br><br>*Evaluator may prompt candidate to identify any significant or notable injury findings*		
**18. Control of hemorrhagic cardiac injury**<br>Correct: Places foley balloon, staples, or suture with pledgets for cardiac wound.		
**19. Identify the thoracic aorta**<br>Correct: Must contain two components:<br>A) <u>Determination of structure:</u> Model correct description includes "I placed my hands posteriorly in the chest cavity, following the ribs until I met the vertebral bodies, then advanced anteriorly and the first structure that I found was the aorta."<br>B) <u>Distinction from esophagus:</u> Correct description includes either "Aorta is the thicker walled structure" or if NG tube requested/placed, "the structure without the tube."<br><br>*Evaluator may prompt candidate "I see your hands in the chest. What are you looking for and how will you know that you have found that structure?"*		
**20. Cross-clamp the thoracic aorta**		
**21. Initiate open cardiac massage**<br>Correct: Places palms together at the base of the heart and gently compresses.		
**22. Maintain sterility throughout procedure**		

Miscellaneous Comments:
