# Supplementary material for: Creation and Implementation of a Mastery Learning Curriculum for Emergency Department Thoracotomy
Source: West J Emerg Med. 2020 Aug 24;21(5):1258–65. doi: 10.5811/westjem.2020.5.46207 (PMC7514402; doi:10.5811/westjem.2020.5.46207)
Supplement: Supplementary file 2 [file wjem-21-1258-s002.docx]

**Appendix 2**

Video of Procedure of Emergency Department Thoracotomy (mp4 file uploaded separately)

<https://vimeo.com/302936118/81371548e3>
